# Diagnostic Challenges in a Case of Immune-Mediated Thrombotic Thrombocytopenic Purpura With Severe ADAMTS13 Deficiency

**DOI:** 10.7759/cureus.67138

**Published:** 2024-08-18

**Authors:** Karel Escoto-Pineda, César Alas-Pineda, Dennis Javier Pavón-Varela, David Cortés

**Affiliations:** 1 Internal Medicine, Dr. Mario Catarino Rivas National Hospital, San Pedro Sula, HND; 2 Medicine and Surgery, Catholic University of Honduras – San Pedro and San Pablo Campus, San Pedro Sula, HND; 3 Internal Medicine, Honduran Social Security Institute, San Pedro Sula, HND

**Keywords:** case report, acute kidney injury, adamts13 protein, von-willebrand factor, thrombotic microangiopathies, thrombotic thrombocytopenic thrombocytopenia

## Abstract

Thrombotic Thrombocytopenic Purpura (TTP) is rare and potentially life-threatening thrombotic microangiopathy (TMA) caused by acquired immune-mediated or congenital deficiency of the von Willebrand factor regulatory enzyme, a Disintegrin And Metalloproteinase with a Thrombospondin Type 1 motif, member 13 (ADAMTS13) which cause microthrombi to form and occlude the microvasculature. The occurrence of acute kidney injury (AKI) in TTP is rare and often underestimated due to confusion with hemolytic uremic syndrome (HUS). A 23-year-old Mestizo male patient presented with altered mental status, hemolytic anemia, thrombocytopenia, intermittent fever, laboratory tests suggestive of thrombotic microangiopathy, and clinical findings consistent with acute kidney injury. Predictive values of the platelet count, lactate dehydrogenase, absent active cancer, schistocytes, mean corpuscular volume, international normalized ratio, creatinine (PLASMIC) score, were used to assess the likelihood of ADAMTS13 deficiency, were employed, and enzymatic activity testing confirmed severe protein deficiency. Honduras' lack of advanced diagnostic capabilities is underscored, emphasizing the urgent need to invest in precision medical technology. ADAMTS13 testing allows for a more precise diagnosis of TTP, which is crucial for early diagnosis and timely treatment.

## Introduction

Thrombotic Thrombocytopenic Purpura (TTP) is a rare and potentially life-threatening thrombotic microangiopathy (TMA) that can be caused by an acquired immune-mediated or congenital deficiency of the von Willebrand factor regulatory enzyme, ADAMTS13 (a disintegrin and metalloproteinase with a thrombospondin type 1 motif, member 13) which cause microthrombi to form and occlude the microvasculature [1]. It is characterized by the development of hemolytic anemia, thrombocytopenia, fever, neurological involvement, and, in rare cases, deterioration of renal function, corresponding to the classic pentad, which is present in only 10% of cases [1,2]. The annual incidence of TTP ranges between 1.5 and six cases per million adults per year, with a prevalence of 10 to 15 cases per million. Additionally, women are predominant with a 2:1 ratio and a higher incidence before the age of 50 [3].

Although renal involvement manifested by proteinuria and hematuria is common in TTP, progression to renal injury is uncommon [2]. Recent studies suggest that acute renal failure (ARF) occurs more significantly in hemolytic uremic syndrome (HUS) within the spectrum of TMAs [1]. TTP results from a marked reduction in the activity of the ADAMTS13 protein [4]. This severe deficiency of ADAMTS13 leads to the accumulation in the blood of ultra-large and highly platelet-adhesive Von Willebrand Factor (vWF) multimers. These persistent multimers contribute to the formation of platelet-rich microthrombi in small-caliber arterioles and capillaries, which in turn induces widespread microvascular ischemia [5].

TTP has been clinically linked to various conditions, including autoimmune diseases, infections, hematopoietic cell and solid organ transplants, neoplasms, the use of certain medications, vaccinations, and pregnancy [6]. It is a medical emergency with potentially life-threatening complications and a mortality rate of 90% if not diagnosed and treated promptly [4-7].

## Case presentation

A 23-year-old Mestizo male patient with altered consciousness, disorientation aggressive behavior, and seizures presented to the Honduras Social Security Institute. On admission, the patient was noted to have a fever, Tmax 38.5 degrees Celsius, accompanied by headache, predominantly bifrontal with a severe intensity. Physical examination revealed generalized pallor, petechiae, and extensive bruises on the forearms. The patient experienced a sudden episode of psychomotor agitation with temporal and spatial disorientation, leading to hospitalization. No significant medical, surgical, or family history is presented. He denies the use of alcohol, tobacco, or any other illegal substances.

**Figure 1:**
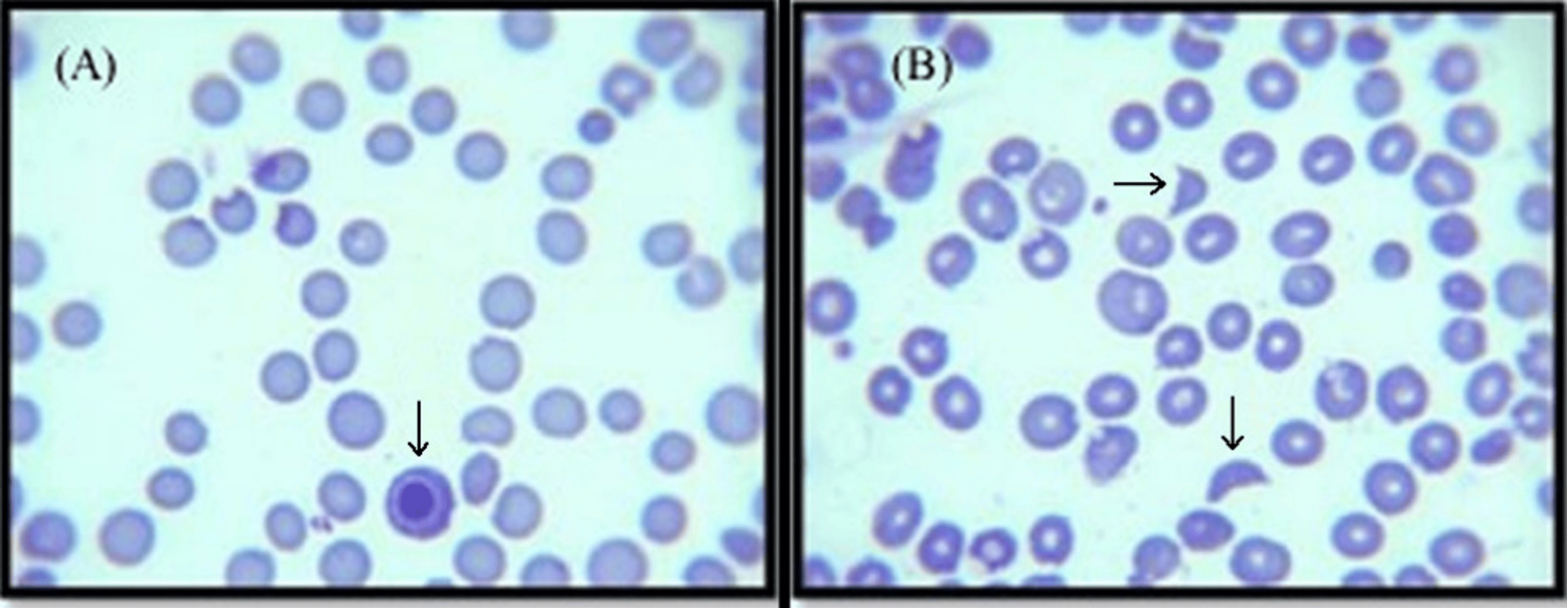
Peripheral blood smear (A) showing polychromasia and severe thrombocytopenia (B) showing schistocytes.

Laboratory findings reported hemoglobin of 8.5 g/dL (range: 14.0-18.0), hematocrit of 24.1% (range: 41-50), elevated corrected reticulocyte count of 6.1% (range: 0.5%-2.5%), and platelet count 14 x 10^9^/L (range: 150-450), white blood cell count of 29.600 x 10^9^/L (range: 4.8-10.8), neutrophils 20.700 x 10^9^/L (range: 1.500-8.500), indirect bilirubin of 2.0 mg/dL (range: 0.2-1.2), and elevated lactate dehydrogenase (LDH) levels > 1000 U/L (range: 312-618) despite a negative Coombs test. Renal function enzymes revealed a serum creatinine concentration of 1.4 mg/dl (range: 0.44 -1). Subsequently, a peripheral blood smear (PBS) demonstrated the presence of five schistocytes per high power field, polychromatophilia, and severe thrombocytopenia corresponding to microscopic findings of microangiopathic hemolytic anemia (Figure 1).

**Figure 2:**
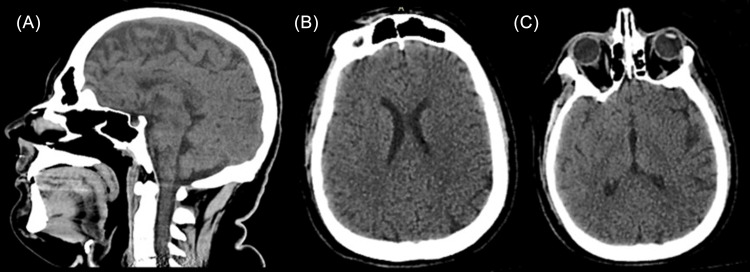
Brain computed tomography (CT) scan with no evident pathologies. (A) Sagittal view of the brain with visible structures and normal appearance. (B,C) Axial views of the brain tissue appears symmetrical without any signs of masses, hemorrhage, or edema.

Additional studies, including tests for cytomegalovirus (CMV) and antibodies against hepatitis C virus (HCV), were negative. Furthermore, antinuclear antibodies (ANA) tests were negative, and complement C3 and C4 values remained within normal ranges. Imaging studies, such as abdominal ultrasound and brain computed tomography (CT) scan, showed no abnormalities (Figure 2). However, due to the absence of a definitive diagnosis, antibiotic and antiviral coverage was initiated due to suspicion of CNS infections (Vancomycin 20mg/kg/day, Ceftriaxone 2g every 12 hours, and Acyclovir 800mg every eight hours).

The predictive values of the platelet count, lactate dehydrogenase, absent active cancer, schistocytes, mean corpuscular volume, international normalized ratio, creatinine (PLASMIC) score were utilized to predict severe acquired ADAMTS13 deficiency; a score of six was obtained (Table 1). Nevertheless, measurement of ADAMTS13 activity levels is not performed in any national diagnostic center in Honduras, samples were sent to the United States for ADAMTS13 evaluation. On day five, the patient experienced bradylalia, bradyphrenia, and increased temporal and spatial disorientation, followed by a generalized tonic-clonic seizure without sphincter relaxation. Severe thrombocytopenia prevented lumbar puncture. Despite the follow-up brain CT scan showing no abnormalities, it was decided to initiate treatment with methylprednisolone pulses (1g per day). Evidence of active bleeding from oral and nasal mucosa, as well as macroscopic hematuria, is noted.

**Table 1:** PLASMIC Score criteria with patient score calculation MCV: mean corpuscular volume, INR: international normalized ratio, PLASMIC: platelet count, lactate dehydrogenase, absent active cancer, schistocytes, mean corpuscular volume, international normalized ratio, creatinine.

Criteria	Score	Patient Value	Score Assigned
Platelet count ≤30 x 10⁹/L	1	14 x 10⁹/L	1
Hemolysis reticulocyte count >2.5%, haptoglobin undetectable, or indirect bilirubin >2.0 mg/dL	1	Reticulocyte count 6.1%, indirect bilirubin 2.0 mg/dL	1
Active cancer	0	No active cancer	1
History of solid-organ or stem-cell transplant	0	None	1
MCV <90 fL	1	50 fL	1
INR <1.5	1	1.5	0
Creatinine <2.0 mg/dL	1	1.4 mg/dL	1
Total PLASMIC Score			6

On day seven, arterial blood gas analysis indicated severe imbalances, with a pH of 7.03, PCO2 of 73.4 mmHg, PO2 of 90.2 mmHg, HCO3 of 19, and elevated lactate at 13.7, indicating type II respiratory failure. Orotracheal intubation was performed using rapid sequence induction without complications. The patient was transferred to the intensive care unit (ICU) due to his poor general condition, where he was placed on mechanical ventilation with vasopressor support. Alveolar and cerebral bleeding were ruled out.

On day 21, the analysis of the ADAMTS13 assay reported a quantified activity of 0.03 IU/mL (range: 0.68-1.63) and an increase in ADAMTS13 inhibitor 9.2 IU/mL (range: <0.4), confirming the acquired deficiency of the enzyme, which provided a definitive diagnosis for severe ADAMTS13 deficiency. Seven sessions of therapeutic plasma exchange (TPE) with a total of 3000 cc per exchange were prescribed as the first-line treatment, and simultaneously, Rituximab 375 mg/m^2^ IV weekly for four weeks was initiated, despite being considered a second-line option and its effects manifesting in the long term. The start of TPE was delayed because the hospital did not have that service available, so it was decided to subcontract the procedure. 

Within the first month of admission, the third hemogram reported hemoglobin of 7.0 g/dL (range: 14.0-18.0), hematocrit of 23.1% (range: 41-50) white blood cell count was reported as 28.000 x 10^9^/L (range: 4.8-10.8), platelets were 7 x 10^9^/L (range: 150-450), and Procalcitonin >100ng/dl (range: <0,05 ng/mL) suspicious for infectious etiology. The patient went into septic shock due to Klebsiella pneumonia, confirmed by a positive blood culture, urine culture, and sputum culture. He was treated with crystalloids and norepinephrine as vasopressor support. Treatment required escalation to Polymyxin B 100 mg IV every 12 hours for 10 days, Fluconazole 200 mg IV daily for 14 days, and Tigecycline 50 mg IV every 12 hours. After the resolution of the septic condition, Prednisone 50 mg orally for 15 days and Rituximab 675 mg in 500 cc of 0.9% NSS were initiated. This corresponds to the second dose of Rituximab because it was prescribed before plasma exchange.

The patient was discharged in good general condition (Table 2), with a Glasgow Coma Scale score of 15, with instructions for oral anticoagulation which involved Enoxaparin 1 mg/kg SC every 12 hours for 48 hours, followed by Apixaban 5 mg PO every 12 hours and a follow-up appointment scheduled in one week for the third dose of Rituximab. However, due to inappropriate adherence to the prescribed anticoagulation regimen, he experienced a symptomatic relapse within one week following his initial discharge. This relapse occurred two months after admission. Popliteal and distal femoral thrombosis were observed on Doppler ultrasound, prompting readmission. Upon evaluation for relapse, evidence of schistocytes, mild thrombocytopenia, and elevated LDH levels were observed again. The treatment regimen was reinitiated, maintaining the same dosing protocol as previously recommended. Glucocorticoid therapy was adjusted to 1 mg/kg for 15 days and the patient's adherence to the therapeutic regimen facilitated a satisfactory recovery. Currently, the patient is receiving follow-up care in outpatient consultation and is in remission.

**Table 2:** Clinical and Laboratory data of patient’s clinical evolution Overview of the patient's clinical evolution with respect to vitals signs, hematological, renal, and additional parameters. BUN: blood urea nitrogen

Category	Parameter	Initial Value	Discharge Value	Normal Range
Vital Signs	Blood Pressure	140/90 mmHg	120/80 mmHg	90/60 - 120/80 mmHg
	Heart Rate	105 bpm	72 bpm	60-100 bpm
	Pulse	105 bpm	72 bpm	60-100 bpm
	Respiratory Rate	22 bpm	17 bpm	12-20 bpm
	Oxygen Saturation	98%	98%	95-100%
	Temperature	38.5°C	36.9°C	36.1°C - 37.2°C
Hematological Parameters	Hemoglobin (Hb)	8.5 g/dL	14.2 g/dL	14.0-18.0 g/dL
	Hematocrit (Hct)	24.1%	42%	41-50%
	Platelet Count	14 x 10⁹/L	180 x 10⁹/L	150-450 x 10⁹/L
	White Blood Cell Count	29.6 x 10⁹/L	8.0 x 10⁹/L	4.8-10.8 x 10⁹/L
	Neutrophils	20.7 x 10⁹/L	5.5 x 10⁹/L	1.5-8.5 x 10⁹/L
	Lymphocytes	2.3 x 10⁹/L	2.3 x 10⁹/L	1.5-5.5 x 10⁹/L
Renal Function	Serum Creatinine	1.4 mg/dL	1.0 mg/dL	0.44-1.0 mg/dL
	BUN	21 mg/dL	15 mg/dL	7-20 mg/dL
Additional Relevant Parameters	Lactate Dehydrogenase (LDH)	1158 U/L	500 U/L	312-618 U/L
	Indirect Bilirubin	2.0 mg/dL	0.8 mg/dL	0.2-1.2 mg/dL
	Reticulocyte Count	6.1%	1.5%	0.5-2.5%

This case underscores the importance of early diagnosis and aggressive management in patients with TTP associated with severe ADAMTS13 deficiency, emphasizing the effectiveness of therapeutic plasma exchange and Rituximab in treating this rare condition.

## Discussion

Classically, TTP is recognized by the pentad of fever, microangiopathic hemolytic anemia, thrombocytopenia, neurologic abnormalities, and in rare cases deterioration of renal function [2,5-7]. Before the availability of ADAMTS13 activity assays, renal involvement served as the primary criterion to differentiate TTP from HUS. This practice resulted in a significant underreporting of TTP cases presenting with severe acute kidney injury (AKI) [3,8,9]. Renal involvement with proteinuria and hematuria are commonly observed in TTP. These findings are consistent with our results, demonstrating clinical signs of AKI evidenced by a serum creatinine concentration of 1.4 mg/dL and macroscopic hematuria. However, the consensus holds that in TMAs, severe acute kidney injury is a hallmark of (HUS), whereas it is less commonly associated TTP and typically observed only through biopsy [8].

Currently, TTP can be classified into congenital TTP (cTTP) or more commonly seen acquired immune-mediated TTP (iTTP) [5,10]. However, the literature reports a higher prevalence of acute kidney injury in patients with cTTP compared to patients with iTTP [3,10,11]. A plausible explanation could be that, unlike patients with cTTP, patients with iTTP retain significant local production of ADAMTS13 in the kidney, which could partially protect against glomerular microangiopathy [3]. Nevertheless, several studies have linked AKI secondary to TTP with severe deficiency of the ADAMTS13 protein [5,10,12]. Multiple studies describe that patients with acute kidney injury induced by iTTP have lower serum levels of C3 than patients with iTTP without acute kidney injury during the acute period [3,9]. However, these findings do not match the normal values of complement C3 and C4 observed in our patient. According to Joly et al., this could be due to dysregulation of the alternative complement pathway [5].

In 2019, Zhao et al. proposed that reduced ADAMTS13 activity leads to the presence of large von Willebrand factor conglomerates in plasma, resulting in the generation of platelet-rich microthrombi in arterioles. Subsequently, schistocytes form as red blood cells traverse the thrombus network, showing a significant increase in LDH levels, even in the early stages of the disease [13].

When assessing the range of patients presenting with thrombotic microangiopathy, the ability to identify cases of thrombotic thrombocytopenic purpura at early stages rapidly is crucial to identify potential clinical outcomes and initiate timely treatment to prevent fatal complications [14]. Nevertheless, the extended turnaround times required for ADAMTS13 activity testing make this assay unsuitable for a real-time clinical decision-making process, especially in developing countries like Honduras, where ADAMTS13 activity test is unavailable. Generally, clinicians in this context manage patients with thrombotic microangiopathy with hemoderivated and make their clinical decisions leaning on clinical and laboratory data.

Different scores use clinical data to predict the risk of thrombotic microangiopathy with and without severe ADAMTS13 deficiency [15,16]. However, the PLASMIC score has proven to be more practical and effective in distinguishing patients who will benefit from TPE until the ADAMTS13 test result comes out [6]. According to Bendapudi et. al. (2017), based on the criteria of the PLASMIC score, laboratory findings suggestive of a severe ADAMTS13 deficiency should report platelet count <30 x 10^9^/L; MCV <90fL; absence of active cancer; absence of stem-cell transplant or solid-organ transplant; combined hemolysis variable assessed by reticulocyte count >2.5% or indirect bilirubin >2.0 mg/dL; INR <1.5; and creatinine <2.0 mg/dL where each finding corresponds to one score [6]. The PLASMIC score was performed in our patient, and a score of six was obtained corresponding to a HIGH (≥90%) pretest probability of TTP (PLASMIC Scores ≥6) which orientated clinical decision-making to begin early management strategy [15].

TPE in conjunction with glucocorticoids, Rituximab, and Caplacizuamb has significantly reduced the mortality and morbidity rates in iTTP [10]. TPE alone can be a life-saving procedure and should be provided to all patients with a suspected TTP [17]. However, the initiation of TPE in our patient was postponed due to the unavailability of the service at the hospital. Subsequently, immunosuppressive therapy with glucocorticoids is fundamental in the management of acute iTTP, the primary objective of this treatment is to inhibit antibody production, facilitating the restoration of circulating ADAMTS13 levels [12,18].

In cases of severe organ failure in the acute phase of PTT, high-dose glucocorticoid therapy as an adjunctive treatment decreases ADAMTS13 antibody body production, and increases the rate of patients achieving complete remission [3]. Furthermore, the International Society on Thrombosis and Haemostasis (ISTH) guidelines recommend Rituximab and caplacizumab ought to be contemplated for patients exhibiting a high likelihood of TTP and those for whom ADAMTS13 test results are attainable [17], in this case, the patient was not treated with caplacizumab because there is no such monoclonal antibody in Honduras.

Various studies describe Rituximab as part of the first-line treatment for severe TTP, due to its ability to accelerate recovery and decrease the incidence of exacerbations and relapses [3,4,10]. However, it is recommended to initiate Rituximab one day after TPE, as its therapeutic action may take several weeks [4,19]. Based on this evidence, the patient is managed with seven sessions of therapeutic plasma exchange of 3000 cc total volume in each exchange and Rituximab 375 mg/m^2^ IV weekly for four weeks, showing relapse two months after completing plasma exchange.

A complete response to treatment is described as the normalization of platelet count (>150 x 10^9^/L) for two consecutive days. A durable response, or clinical remission, is considered when this normalization persists for at least 30 days after completion of TPE [10,17]. Clinical exacerbation corresponds to a platelet count (<150 x 10^9^/L) within 30 days of discontinuation of plasma exchange treatment. Recurrent disease occurs if symptoms reappear 30 or more days after achieving treatment response. In the case of refractory TTP, it is characterized by the lack of sustained response to treatment up to day 60 [4,5,10,11].

Recently, Cuker et al. described partial remission of ADAMTS13 as ADAMTS13 activity ≥20% but less than the lower limit of normal (LLN), and complete remission as ADAMTS13 activity ≥ LLN [20]. Clinical relapse of TTP is described as a platelet count (<150 x 10^9^/L) with or without evidence of ischemic organ injury after clinical remission [20]. The same author differentiated clinical relapse from ADAMTS13 relapse, recognizing that an ADAMTS13 relapse (decrease in activity <20% after partial or complete remission of ADAMTS13) can occur without clinical relapse [20]. During the clinical relapse evaluation of our patient, schistocytes, mild thrombocytopenia, and elevated LDH levels were noted again. The glucocorticoid therapy was adjusted to 1 mg/kg for 15 days, and a new Rituximab dose was added, which resulted in remission for the patient. The patient is now being followed up in outpatient consultations.

Previously, mortality from TTP was around 90%; however, this figure has decreased over time due to the effectiveness of TPE [12]. According to the British Society of Hematology, daily plasma exchange, usually with rotating apheresis, reduces mortality from 90% to 10%-20%, replenishes ADAMTS13, and eliminates autoantibodies [10]. Recently, Okoli et al. suggested a daily exchange session of 60 ml/kg plasma volume (1.5 times the estimated plasma volume) until the platelet count remains above (>150 x 10^9^/L) for at least 48 hours, which corresponds to a complete response to treatment [4]. On the other hand, two sessions per day should be considered in refractory TTP or when there is no response to the initial dose [5,10].

This case highlights the importance of recognizing the syndromic presentation of thrombotic microangiopathy and the early diagnosis of thrombotic thrombocytopenic purpura for appropriate management, as immediate treatment can make a difference in clinical outcomes for these patients. It is crucial to highlight that, currently, routine measurement of ADAMTS13 activity levels is not performed in any national diagnostic center in Honduras. The urgency of sending samples to the United States for ADAMTS13 evaluation underscores the need to develop more advanced diagnostic capabilities in the country, which would undoubtedly facilitate the identification of potential cases at early stages. This, in turn, could have a significantly positive impact on clinical outcomes and the quality of life of affected individuals.

## Conclusions

TTP represents a medical emergency due to its risk of organ damage and potentially fatal complications. The occurrence of acute kidney injury in TTP is often underestimated due to confusion between TTP and HUS. The ADAMTS13 test enables a more precise diagnosis of TTP, which is necessary for early diagnosis and timely treatment.

## References

[1] Mingot Castellano ME, Pascual Izquierdo C, González A (2022). Recommendations for the diagnosis and treatment of patients with thrombotic thrombocytopenic purpura. Med Clin (Barc).

[2] Najar H, Tuider L, Kukkar V, Quasem M (2022). Thrombotic thrombocytopenic purpura: a rare cause of severe acute kidney injury. Cureus.

[3] Fodil S, Zafrani L (2022). Severe thrombotic thrombocytopenic purpura (TTP) with organ failure in critically ill patients. J Clin Med.

[4] Okoli S, Jenkins KA, Bojanowski CM (2023). Current intensive care management of thrombotic thrombocytopenic purpura: a case report and updated literature review. J Intensive Care Med.

[5] Joly BS, Coppo P, Veyradier A (2019). An update on pathogenesis and diagnosis of thrombotic thrombocytopenic purpura. Expert Rev Hematol.

[6] Bendapudi PK, Li A, Hamdan A (2015). Impact of severe ADAMTS13 deficiency on clinical presentation and outcomes in patients with thrombotic microangiopathies: the experience of the Harvard TMA Research Collaborative. Br J Haematol.

[7] Nuñez Matamoros G, Delgado Carreño J, Navas Serrano R, Ladines Castro WJ (2020). Purpura trombocitopénica trombótica: informe de caso clínico (Article in Spanish). RECIMUNDO.

[8] Issa L, Sandakly N, El Koubayati G, Khalil M, Haddad F (2024). Renal involvement in thrombotic thrombocytopenic purpura: is it time to challenge the old paradigm?. Cureus.

[9] Zafrani L, Mariotte E, Darmon M (2015). Acute renal failure is prevalent in patients with thrombotic thrombocytopenic purpura associated with low plasma ADAMTS13 activity. J Thromb Haemost.

[10] Scully M, Rayment R, Clark A (2023). A British Society for Haematology Guideline: diagnosis and management of thrombotic thrombocytopenic purpura and thrombotic microangiopathies. Br J Haematol.

[11] Scully M, Cataland S, Coppo P (2017). Consensus on the standardization of terminology in thrombotic thrombocytopenic purpura and related thrombotic microangiopathies. J Thromb Haemost.

[12] Long B, Bridwell RE, Manchanda S, Gottlieb M (2021). Evaluation and management of thrombotic thrombocytopenic purpura in the emergency department. J Emerg Med.

[13] Zhao N, Zhou L, Hu X (2020). A modified PLASMIC score including the lactate dehydrogenase/the upper limit of normal ratio more accurately identifies Chinese thrombotic thrombocytopenic purpura patients than the original PLASMIC score. J Clin Apher.

[14] Bendapudi PK, Hurwitz S, Fry A (2017). Derivation and external validation of the PLASMIC score for rapid assessment of adults with thrombotic microangiopathies: a cohort study. Lancet Haematol. Published Online First.

[15] Bentley MJ, Lehman CM, Blaylock RC, Wilson AR, Rodgers GM (2010). The utility of patient characteristics in predicting severe ADAMTS13 deficiency and response to plasma exchange. Transfusion.

[16] Coppo P, Schwarzinger M, Buffet M (2010). Predictive features of severe acquired ADAMTS13 deficiency in idiopathic thrombotic microangiopathies: the French TMA reference center experience. PLoS One.

[17] Zheng XL, Vesely SK, Cataland SR (2020). ISTH guidelines for the diagnosis of thrombotic thrombocytopenic purpura. J Thromb Haemost.

[18] Sukumar S, Lämmle B, Cataland SR (2021). Thrombotic thrombocytopenic purpura: pathophysiology, diagnosis, and management. J Clin Med.

[19] Owattanapanich W, Wongprasert C, Rotchanapanya W, Owattanapanich N, Ruchutrakool T (2019). Comparison of the long-term remission of rituximab and conventional treatment for acquired thrombotic thrombocytopenic purpura: a systematic review and meta-analysis. Clin Appl Thromb Hemost.

[20] Cuker A, Cataland SR, Coppo P (2021). Redefining outcomes in immune TTP: an international working group consensus report. Blood.

